# Metagenome-Assembled Genome Sequence of a Strain of Burkholderia cepacia Isolated from the Gut of Macrotermes bellicosus in Nigeria

**DOI:** 10.1128/mra.00777-22

**Published:** 2023-01-04

**Authors:** Jessica L. Lenka, Bitrus Yakubu, Richard J. Kutshik, Enoch B. Joel, Aminu Tukur, Ishaya Y. Longdet

**Affiliations:** a Department of Biochemistry, University of Jos, Jos, Nigeria; b Biotechnology Centre, National Veterinary Research Institute, Vom, Nigeria; c Quality Control Department, Nigeria National Petroleum Corporation, Port Harcourt Refinery, Port Harcourt, Nigeria; University of Maryland School of Medicine

## Abstract

The efficiency of the termite Macrotermes bellicosus at digesting lignocellulose is due to its gut bacterial symbionts. We report the metagenome-assembled genome sequence of Burkholderia cepacia UJ_SKK_1.2, reconstructed from metagenomes produced from *Macrotermes bellicosus* gut microbiota. The 7,460,271-bp genome obtained consists of 6,763 protein-coding sequences, with 6,719 functionally assigned genes and 59 RNA genes.

## ANNOUNCEMENT

*Burkholderia* species occupy diverse ecological niches (1) and have many uses, including for bioremediation, lignocellulose degradation, pesticides, biofertilizers, natural product discovery, and plant growth promotion (2–6). Here, we present the genome sequence of a strain of Burkholderia cepacia recovered from Macrotermes bellicosus termites, which digest lignocellulose effectively due to their gut symbiotic bacteria (7–9).

*Macrotermes bellicosus* specimens collected from Illela, Sokoto State, Nigeria (lat 38.973166, long 122.72809), in February 2021 were cleaned 3 times (dipped in 70% ethanol for 3 min and rinsed with sterile water). To select only organisms with the characteristic of interest in this project (lignocellulose degradation), a termite gut was extracted in phosphate-buffered saline (PBS) and crushed; it was cultured on plated medium prepared from Kraft lignin, M9 salts, and agar-agar at a ratio of 2:1:2 at 37°C for 72 h and subcultured in four successions on the same medium. We thought that the colonies were pure isolates, but Gram staining revealed a mixture of cocci and rods appearing either in pairs, clusters, chains, or singly. To obtain cell pellets, the mixed organisms were grown overnight in nutrient broth at 37°C. Two sets of 2-mL portions were centrifuged at 14,000 × *g* for 3 min. The combined cell pellets were washed (in 500 μL phosphate-buffered saline) and centrifuged at 14,000 × *g* for 3 min twice.

DNA extraction of the cells was performed using the ZymoBIOMICS DNA miniprep kit, according to the manufacturer’s instructions. DNA libraries were prepared using the Nextera XT DNA library preparation kit (Illumina) and Nextera index kit (Illumina), and the genomic DNA was fragmented using Illumina Nextera XT fragmentation enzyme. Combinatory dual indexes were added to each sample, followed by 12 cycles of PCR to construct libraries, which were purified using AMPure magnetic beads. The libraries were then eluted in Qiagen elution buffer (EB), quantified using a Qubit 4 fluorometer and Qubit double-stranded DNA (dsDNA) high-sensitivity (HS) assay kit, and sequenced on an Illumina HiSeq X platform with 2 × 150-bp read lengths, producing 7.208 million raw reads. The raw metagenomic sequencing reads were trimmed and processed using Fastp v0.20.1 (10), with a mean cut quality of 15. The reads were assembled into contigs using MEGAHIT v1.0 (11) and then binned using MetaBAT v2 (12), producing 8 hits, but only two metagenome-assembled genomes (ABHB1_5.1 and ABHB1_5.2) were sizably obtained (completeness, ≥50%; contamination, ≤10%) (13). Here, default parameters were used for all software. The taxonomic classification, completeness, and contamination of the ABHB1_5.1 and ABHB1_5.2 genomes were assessed using QUAST v4.4 (14) and BUSCO v5 (15). The ABHB1_5.1 genome sequence was of poor quality. The ABHB1_5.2 genome was of high quality, with 52 contigs (mean, 143,466.75 bp), an assembly size of 7,460,271 bp, completeness of 92.70%, missing portion of 7.30%, *N*_50_ value of 270,795 bp, and a GC content of 67.7%. The BUSCO score was as follows: C:115 (S:114, D:1), F:0, M:9, n:124. The genome was annotated using PGAP v6.1 (16), identifying 6,763 protein-coding sequences, 6,719 proteins with functional assignments, 44 pseudogenes, and 59 RNA genes.

**FIG 1:**
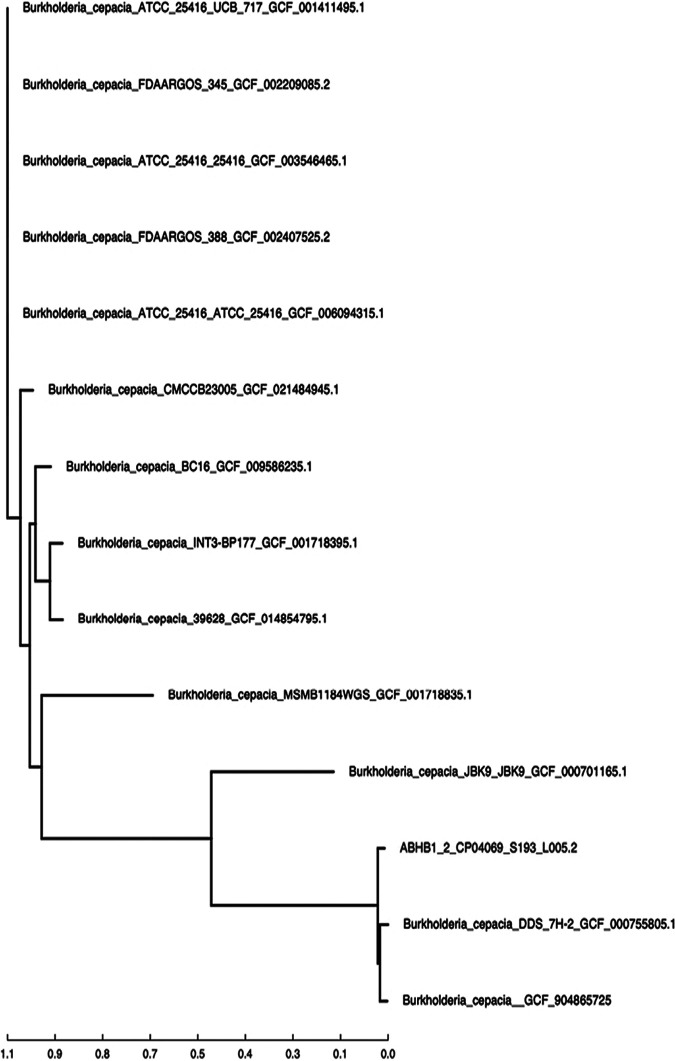
SNP tree based on the core genome phylogeny of ABHB1_5.2 (strain UJ_SKK_1.2). CosmosID’s bacterial database was used to determine the species of the sample. Based on the species ID of the sample, reference genomes were downloaded from NCBI’s RefSeq database to be used in the generation of the SNP tree. The genomes were then processed through the CosmosID core genome SNP typing pipeline to evaluate the phylogenetic placement and SNP differences for meaningful epidemiological inferences. Additional details were determined using Parsnp (17) and FastTree v2 (18).

The assembled contigs were processed through the CosmosID core genome single-nucleotide polymorphism (SNP) typing pipeline to evaluate the phylogenetic placement and SNP differences, using Parsnp (17) as the core genome aligner; the phylogenomic relationships among the genomes were reconstructed using FastTree v2 (18). These relationships were used to generate an SNP tree (Fig. 1), revealing closely related strains of Burkholderia cepacia. Therefore, we named the organism Burkholderia cepacia strain UJ_SKK_1.2.

### Data availability.

The genome data have been deposited at DDBJ/EMBL/GenBank under accession number JAMDXW000000000. The version described in this paper is JAMDXW010000000. The raw reads were deposited in the Sequence Read Archive under accession number SRR19200149.
